# Reappraisal of the Entity of Pediatric Uveitis: Bilateral Iridocyclitis With Retinal Capillaritis (BIRC) in Juveniles in 18 Additional Cases

**DOI:** 10.7759/cureus.112232

**Published:** 2026-07-07

**Authors:** Toshihiko Matsuo

**Affiliations:** 1 Ophthalmology, Graduate School of Interdisciplinary Science and Engineering in Health Systems, Okayama University, Okayama, JPN

**Keywords:** bilateral iridocyclitis with retinal capillaritis (birc), colchicine, corticosteroid, fluorescein angiography, juvenile, juvenile chronic iridocyclitis (jci), juvenile idiopathic arthritis (jia), macular edema, optical coherence tomography, optic disc edema

## Abstract

Objectives: Bilateral iridocyclitis with retinal capillaritis (BIRC) in juveniles is a newly recognized ​​​​​entity of juvenile chronic uveitis with no systemic involvement. In this study, 18 additional consecutive patients with BIRC were reported to show that the entity would be useful in the differential diagnosis.

Methods: Clinical features were retrospectively reviewed in 18 consecutive patients diagnosed with BIRC in juveniles at Okayama University Hospital, Okayama, Japan, between 1996 and 2025.

Results: The 18 patients comprised 6 males and 12 females, with an age at onset ranging from 9 to 16 years (median, 13 years). All patients presented with aqueous cells and mutton-fat keratic precipitates in both eyes, and fluorescein angiography revealed varying degrees of optic disc leakage and retinal capillary leakage in the midperipheral to peripheral fundus of both eyes. None had systemic symptoms, including skin rashes, joint pain, or fever, and no abnormalities were detected on blood examinations, urinalysis, or plain chest x-rays. Oral prednisolone, tapered from 30 mg daily, together with topical 0.1% betamethasone four times daily, was administered to five patients: two with optic disc edema and three with macular edema in both eyes or the unilateral eye. The remaining 13 patients were treated with topical 0.1% betamethasone eye drops alone. In patients who developed elevated intraocular pressure, often due to a steroid response, the instillation of topical 0.1% betamethasone was reduced to twice daily, or intraocular pressure-lowering eye drops were used in combination. In two patients who showed relapse of unilateral macular edema after oral prednisolone was tapered or discontinued, oral colchicine (1 mg daily) was introduced, resulting in the subsidence of macular edema. Bilateral uveitis in all 18 patients subsided within 6 months to a few years until 19 years of age, and all topical medications were discontinued, with relapse occurring in only one patient.

Conclusions: BIRC in juveniles should be included in the differential diagnosis for school-age children and young adolescents. BIRC follows a chronic course of a few years and subsides completely with a good prognosis. In cases of macular edema or optic disc edema that affects vision, oral corticosteroid treatment is recommended, whereas patients with no sight-threatening signs can be treated with topical corticosteroid eye drops alone. The entity of BIRC may be distinct from juvenile chronic iridocyclitis in young girls with onset at preschool age, which shares common features with juvenile idiopathic arthritis-associated uveitis.

## Introduction

Uveitis is the term used to describe inflammatory conditions of both infectious and non-infectious origin that affect intraocular tissues such as the retina, choroid, ciliary body, and iris. The diagnosis of uveitis is based on clinical findings: aqueous cells, keratic precipitates, and iris nodules as signs of anterior uveitis as well as optic disc injection and swelling, retinal vascular leakage, obliteration and sheathing, retinal spots and infiltrates, vitreous opacity, and choroidal nodules and infiltrates as signs of posterior uveitis. In the diagnostic procedure, infectious uveitis, such as syphilis and tuberculosis, is first excluded based on the comprehensive clinical picture, targeted imaging, and laboratory investigations. Non-infectious uveitis is then considered based on the clinical manifestations and laboratory examinations [1-3].

Uveitis in children is rare and difficult to diagnose, as it is sometimes hard to obtain children's cooperation during clinical testing [4]. Major entities in the differential diagnoses of uveitis in children are juvenile idiopathic arthritis [5-7], tubulointerstitial nephritis [8], inflammatory bowel disease [9], autoinflammatory diseases with a genetic background, such as Blau syndrome [10,11], and Behçet disease [12,13], all of which show systemic manifestations in addition to bilateral uveitis or, occasionally, unilateral uveitis at onset. Even more rarely, bilateral uveitis in isolation with no systemic manifestations is noted after systemic diseases are carefully excluded by medical interview and systemic examinations, including blood tests, urinalysis, and plain chest X-ray films.

The well-known entity of isolated bilateral uveitis in childhood, which was established in the 1960s, is juvenile chronic iridocyclitis in preschool-aged girls, which shows ophthalmic features similar to those of juvenile idiopathic arthritis-associated uveitis [14]. The 1997 report by the author described a new entity of isolated bilateral uveitis in school-age children and young adolescents that was called “bilateral iridocyclitis with retinal capillaritis (BIRC)" in juveniles [4] to differentiate it from juvenile chronic iridocyclitis in young girls [14]. In that study, which covered the years from 1985 to 1995, 18 consecutive patients with an age at onset ranging from 9 to 17 years showed aqueous cells and mutton-fat keratic precipitates, together with leakage from the optic disc and retinal capillaries, mainly in the midperiphery of the fundus in both eyes. The uveitis took a chronic course and subsided in a few years [4]. This study reports 18 additional consecutive patients with BIRC seen at the same institution between 1996 and 2025.

## Materials and methods

A retrospective review was conducted on 18 consecutive patients diagnosed with BIRC in juveniles between 1996 and 2024. The latest follow-up was conducted until March 2026. This study was approved by the Ethics Committee of Okayama University Graduate School of Medicine, Dentistry, and Pharmaceutical Sciences and Okayama University Hospital as a retrospective observational study (Identifier: 2403-055; date of approval: March 22, 2024). The data retrieved from the medical records included gender, age at onset, clinical features, topical and systemic treatment, follow-up period, number of relapses, and age at remission.

**Figure 1:**
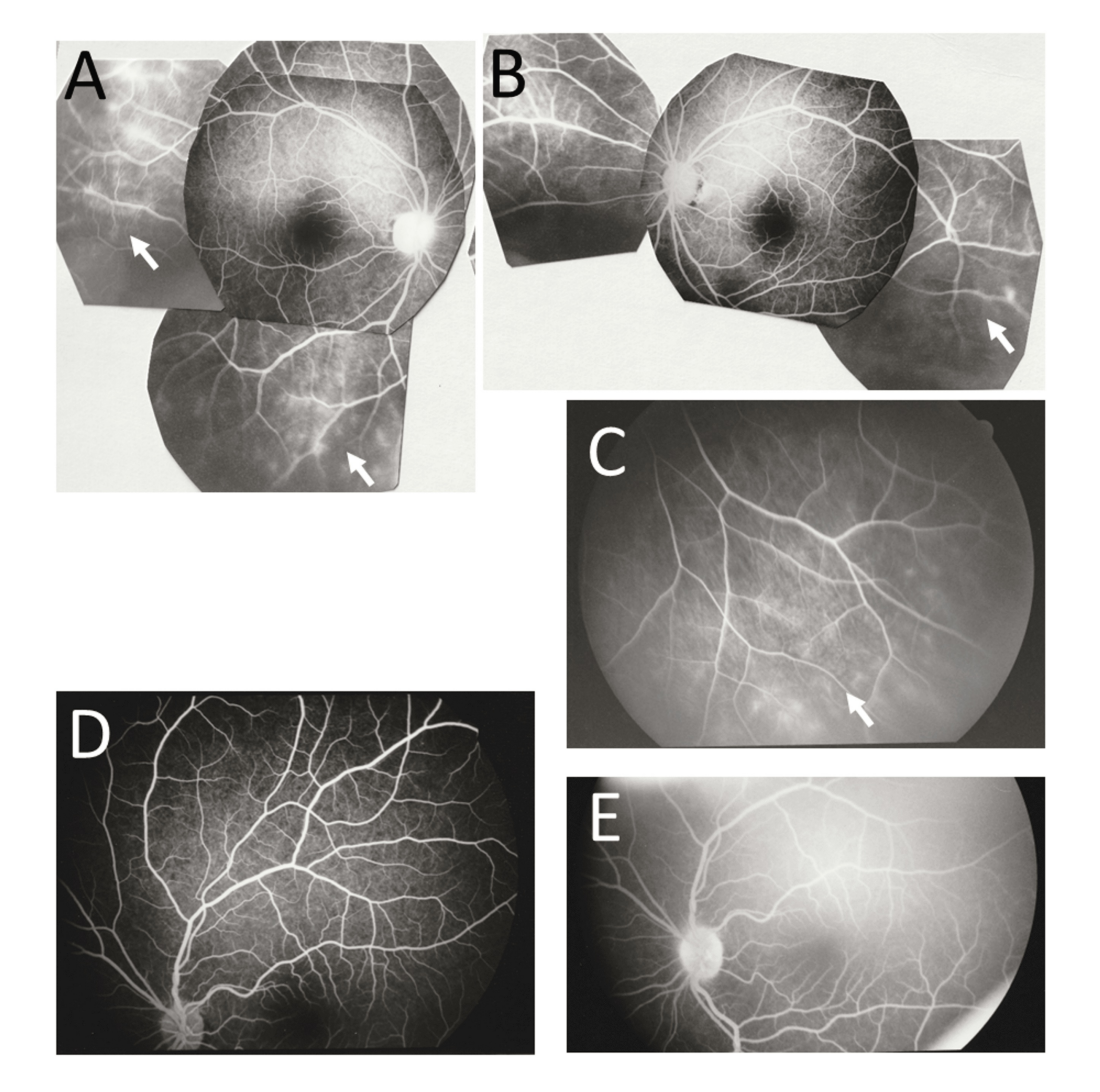
Clinical images in Cases 1, 2, and 6 Case 1: A nine-year-old female. Montage fluorescein angiograms of the right eye (A) and left eye (B) show optic disc leakage with staining and midperipheral retinal capillary leakage (arrows). Case 2: A 12-year-old female. Fluorescein angiography of the left eye (C) shows midperipheral retinal capillary leakage (arrow). Case 6: A 10-year-old female. The late phase of fluorescein angiography of the left eye (E) shows optic disc staining with mild retinal capillary leakage compared with the early phase (D).

**Figure 2:**
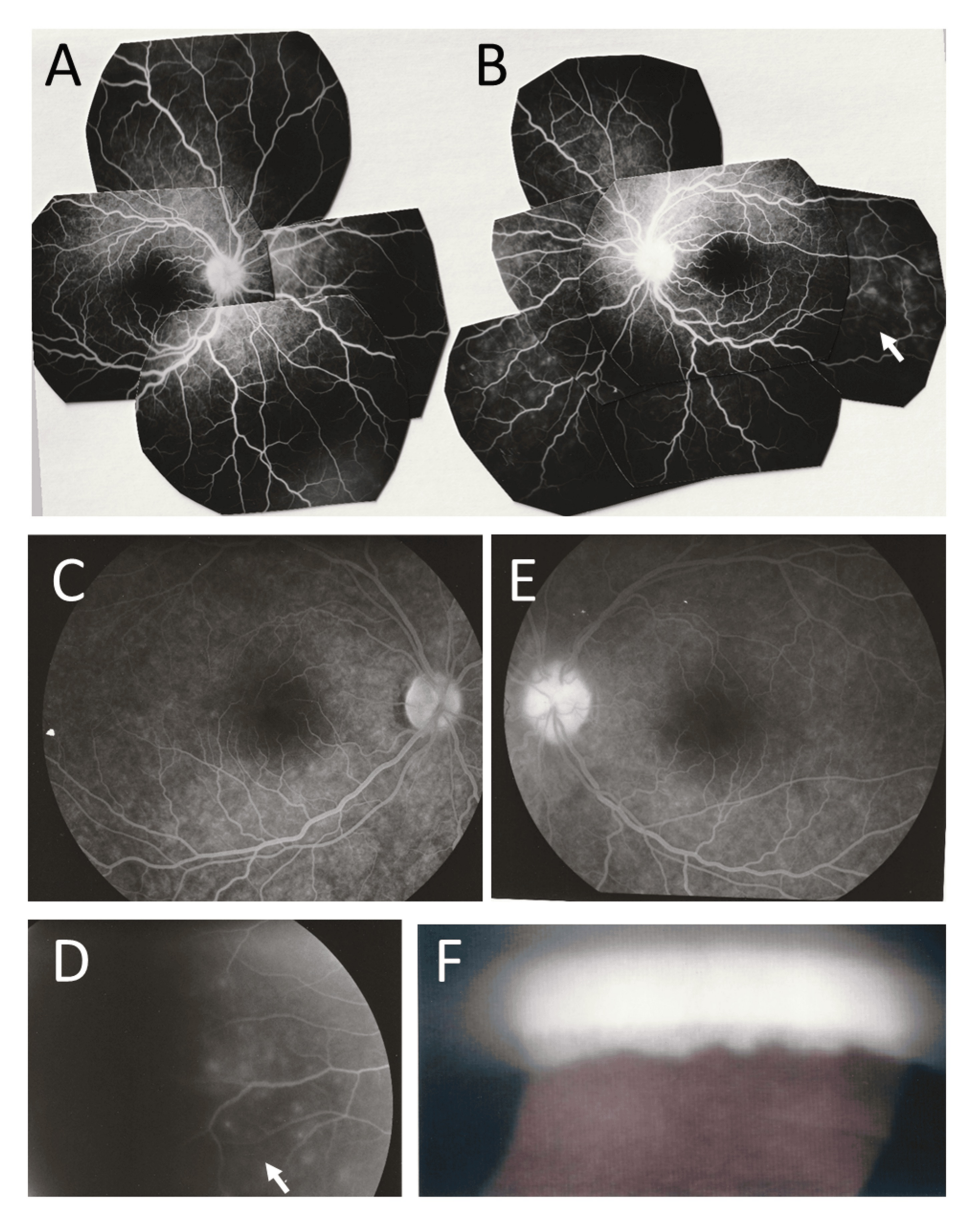
Clinical images in Cases 5 and 11 Case 5: A 14-year-old female. Montage fluorescein angiograms of the right eye (A) and left eye (B) show optic disc leakage with staining and midperipheral retinal capillary leakage (arrow). Case 11: A 16-year-old male. Late-phase fluorescein angiograms of the right eye (C, D) and left eye (E) show optic disc leakage with staining and mild retinal capillary leakage (arrow). Gonioscopy (F) shows tent-like anterior iris synechiae.

The diagnosis of BIRC was made using the same criteria as those described previously [4]. The clinical features included bilateral iridocyclitis, which occurred simultaneously in both eyes or had an onset with a time interval between the two eyes, and retinal cloudiness in the peripheral-to-midperipheral fundus of both eyes with hyperemic optic discs. In all patients, fluorescein angiography showed dye leakage from retinal capillaries to a varying extent, corresponding to retinal cloudiness in the fundus (Figures 1, 2).

Infectious uveitis and non-infectious uveitis associated with systemic diseases were excluded by routine examinations, which included blood examinations, urinalysis, plain chest X-ray films, and tuberculin skin tests in earlier years when interferon-γ releasing assays were not available. Blood examinations included complete blood cell counts, blood chemistry in a common set of liver and renal function tests, electrolytes, cholesterol, C-reactive protein, and glucose. Infectious disease testing included serological tests for syphilis (rapid plasma reagin and treponemal antibodies), hepatitis B virus surface antigen (HBsAg), hepatitis C virus antibody (HCV-Ab), human T-lymphotropic virus type 1 (HTLV-1) antibody, and interferon-γ release assays after they became available. Antinuclear antibody was measured as an immunological test. In urinalysis, total protein, N-acetyl-β-D-glucosaminidase (NAG), and β2-microglobulin were measured in 24-hour urine collections in most patients, whereas they were measured in spot urine samples in recent years. Consultation with pediatricians and rheumatologists was the norm to exclude infectious and non-infectious systemic diseases such as syphilis, tuberculosis, juvenile idiopathic arthritis, tubulointerstitial nephritis, inflammatory bowel disease, and Behçet disease [4].

## Results

Selected case reports

Case 10

**Figure 3:**
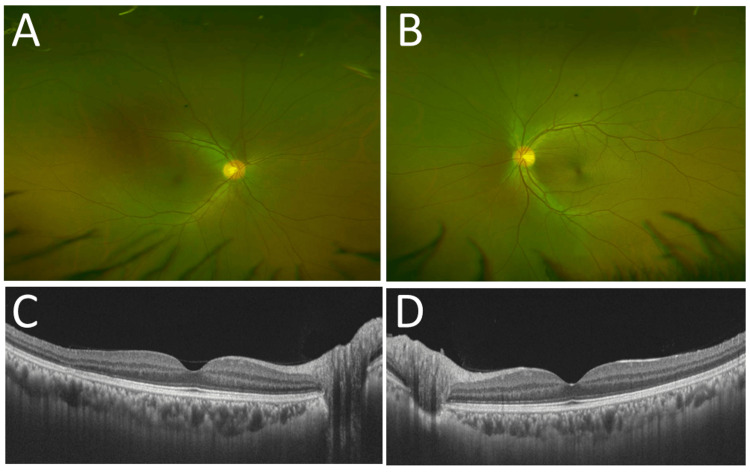
Clinical images in Case 10 Wide-field fundus photographs (right eye: A; left eye: B) and horizontal section images of optical coherence tomography (right eye: C; left eye: D), showing normal discs and maculae in both eyes of a woman at 40 years of age after 27 years of follow-up from disease onset at 13 years of age.

A 13-year-old female noticed redness and blurring in the left eye and was prescribed topical 0.1% betamethasone four times daily for the left eye with a diagnosis of iridocyclitis. At referral, the best-corrected visual acuity was 1.5 in both eyes, and the intraocular pressure was 12 mmHg in both eyes. She showed no inflammation in the right eye and had 1+ mutton-fat keratic precipitates and a few aqueous cells in the left eye. Fluorescein angiography showed mild leakage from the optic discs and midperipheral retinal capillary leakage in both eyes. She had no history, and consultation with a pediatrician revealed no systemic abnormalities. At the age of 17 years, she developed iridocyclitis in the right eye. Repeat urinalysis with 24-hour urine collection again showed no abnormalities. At the age of 19 years, she discontinued the eye drops and subsequently showed no relapse until the latest visit at the age of 43 years (Figure 3).

Case 11

A 16-year-old male complained of pain in the left eye and had been prescribed topical 0.1% betamethasone four times daily with a diagnosis of bilateral iridocyclitis two months previously. He experienced a relapse and was treated with oral prednisolone 30 mg daily for two weeks and then was referred to a university hospital. The best-corrected visual acuity was 1.5 in both eyes, and the intraocular pressure was 16 mmHg in both eyes. Slit-lamp examination showed 1+ mutton-fat keratic precipitates and 1+ aqueous cells in both eyes. The optic discs in both eyes were mildly hyperemic, and fluorescein angiography showed late-phase dye staining of the optic discs as well as midperipheral retinal capillary leakage in both eyes (Figure 2, Panels C-E). He did not have any past medical history. Consultation with a rheumatologist revealed no systemic abnormalities.

In urinalysis, measurements of total protein, N-acetyl-β-D-glucosaminidase (NAG), and β2-microglobulin in a 24-hour urine collection were all negative. Oral prednisolone was tapered and discontinued in two weeks, and topical betamethasone was replaced with 0.1% fluorometholone four times daily because the intraocular pressure increased to 24 mmHg in both eyes. One month later, he experienced a relapse of iridocyclitis with 2+ mutton-fat keratic precipitates and 2+ aqueous cells in both eyes, and oral prednisolone 30 mg daily with topical betamethasone was resumed. The tapering schedule for oral prednisolone at this time was 20 mg daily for two weeks, 30 mg every other day for two weeks, and then 20 mg every other day. After one year, oral prednisolone was discontinued, and he had a small number of keratic precipitates while using topical betamethasone two to four times daily. The intraocular pressure in both eyes remained within the normal range. He was followed for another year with no relapse until the age of 18 years.

Case 12

**Figure 4:**
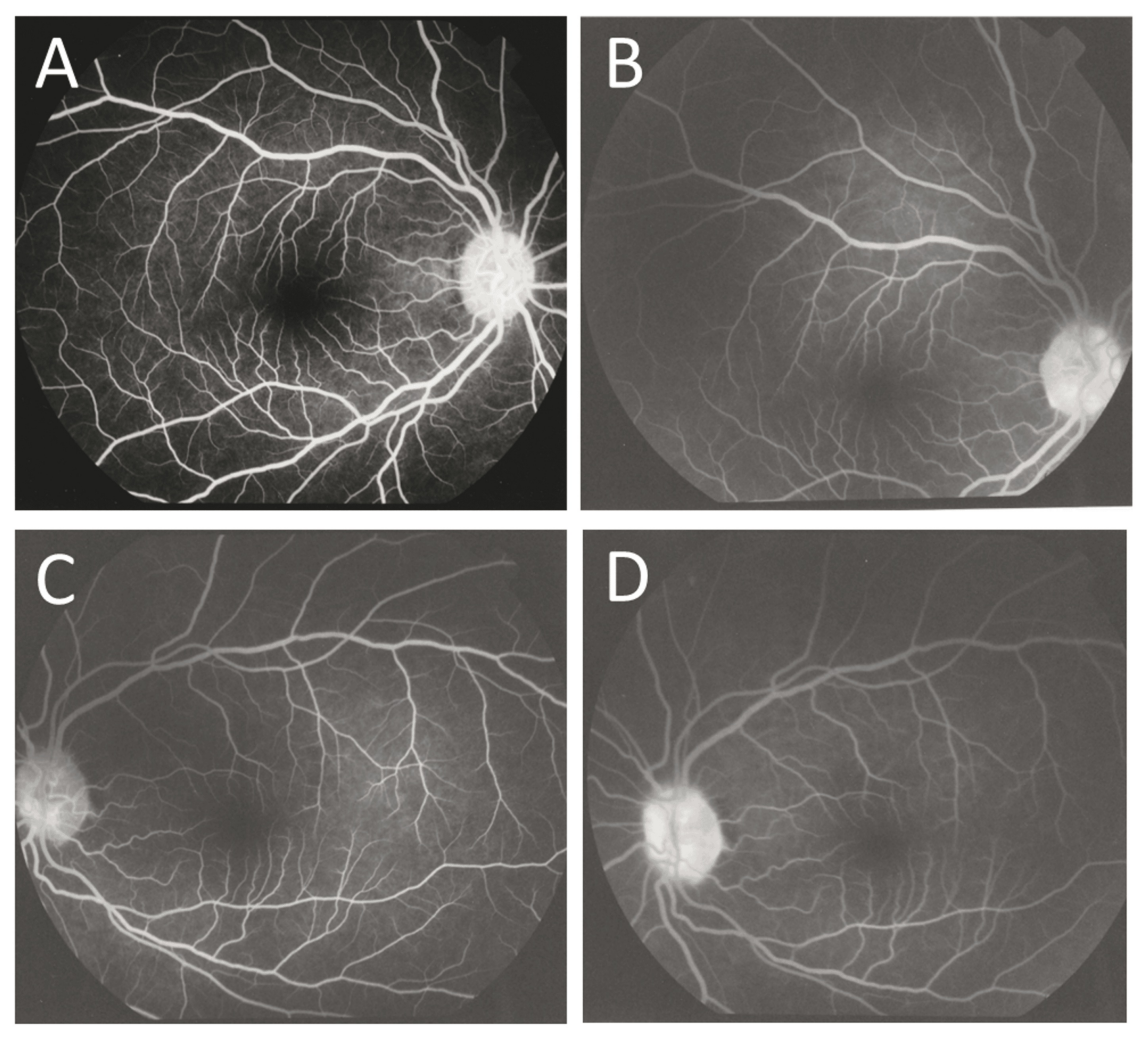
Clinical images in Case 12 A 13-year-old male. Fluorescein angiograms (A, B: right eye; C, D: left eye), showing dye leakage from the optic disc in both eyes in the late phase (B, D) compared with the early phase (A, C).

A 13-year-old male noticed an injection in both eyes half a year previously and used topical 0.1% betamethasone four times daily for iridocyclitis. At referral, the best-corrected visual acuity was 1.5 in the right eye and 1.2 in the left eye. The intraocular pressure was 15 mmHg in both eyes. Slit-lamp examination showed 2+ mutton-fat keratic precipitates and 2+ aqueous cells in both eyes. The optic discs in both eyes were mildly hyperemic, and fluorescein angiography showed late-phase dye staining of the optic discs as well as peripheral retinal capillary leakage in both eyes (Figure 4). He had no relevant medical history, and systemic examinations, including blood examinations, a plain chest X-ray film, and a tuberculin skin test, were all normal. In urinalysis, measurements of total protein, NAG, and β2-microglobulin in a 24-hour urine collection were all negative. He continued topical betamethasone eye drops two to four times daily, depending on the extent of iridocyclitis, over the following four years. He did not show elevated intraocular pressure. Topical betamethasone was then replaced with 0.1% bromfenac twice daily. He did not experience a relapse of iridocyclitis in either eye during a further two years of follow-up until the age of 18 years.

Case 16

**Figure 5:**
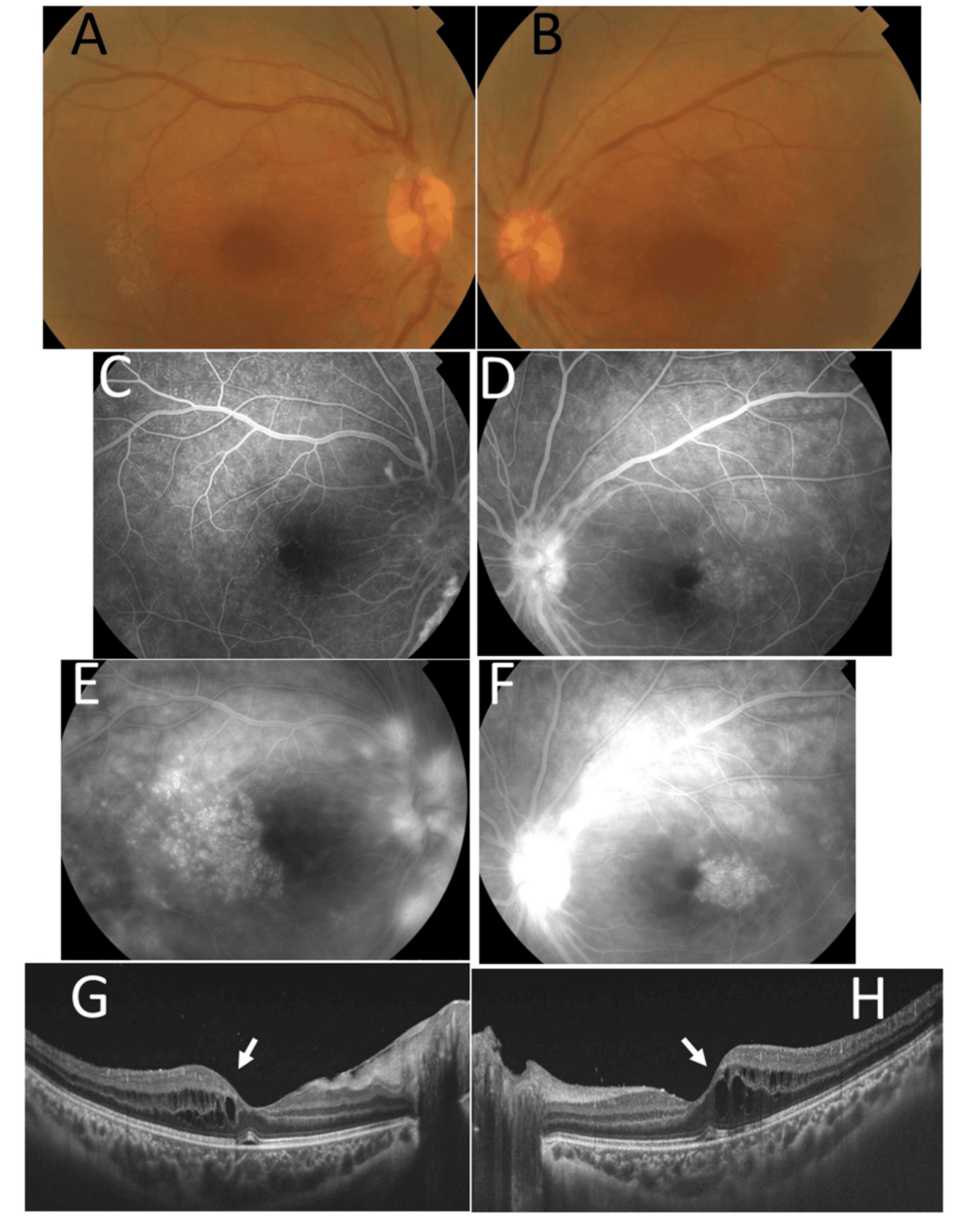
Clinical images in Case 16 (female) at 14 years of age Color fundus photographs (A: right eye; B: left eye), showing optic disc swelling in both eyes. Fluorescein angiographs (C, E: right eye; D, F: left eye), showing marked leakage in the late phase (E, F), compared with the early phase (C, D). Horizontal section images of optical coherence tomography (G: right eye; H: left eye), showing intraretinal fluids on the temporal side (arrows).

A 14-year-old female noticed dyschromatopsia, with yellow appearing white. She was diagnosed with bilateral optic disc edema and referred to a university hospital. The best-corrected visual acuity in decimals was 0.8 in both eyes, and the intraocular pressure was 15 mmHg in both eyes. She showed 1+ mutton-fat keratic precipitates, 1+ aqueous cells, and 1+ vitreous cells, with no iris nodules or iris synechiae in both eyes. The optic discs in both eyes were swollen and hyperemic (Figure 5, Panels A and B), and the maculae in both eyes had cystoid edema. Because optic neuritis was suspected, she was referred to a neurologist, who found no abnormalities. Goldmann perimetry showed normal visual fields with slight enlargement of the Mariotte scotoma in both eyes. Head magnetic resonance imaging showed no abnormalities. A plain chest X-ray film was normal. Blood examinations, including antinuclear antibodies and anti-aquaporin 4, as well as urinalysis, including the measurement of NAG, were all normal. Serological tests for syphilis, hepatitis B virus surface antigen, hepatitis C virus antibody, and human T-lymphotropic virus type 1 (HTLV-1) antibody were all negative. Fluorescein angiography showed dye leakage from the optic discs and retinal capillaries in the late phase, with dye pooling in cystoid spaces temporal to the macula (Figure 5, Panels C-F). Optical coherence tomography revealed cystoid macular edema in both eyes (Figure 5, Panels G and H).

She began oral prednisolone 30 mg daily, which was tapered to 5 mg daily over two months, together with topical 0.1% betamethasone four times daily. Two weeks later, she experienced a relapse of iridocyclitis in the left eye with numerous aqueous cells, fibrin deposition, and ciliary injection. The interview revealed that she was taking oral prednisolone 5 mg daily but was not using topical betamethasone eye drops regularly. She resumed topical betamethasone four to six times daily in both eyes, together with oral prednisolone 5 mg daily, resulting in the subsidence of iridocyclitis.

**Figure 6:**
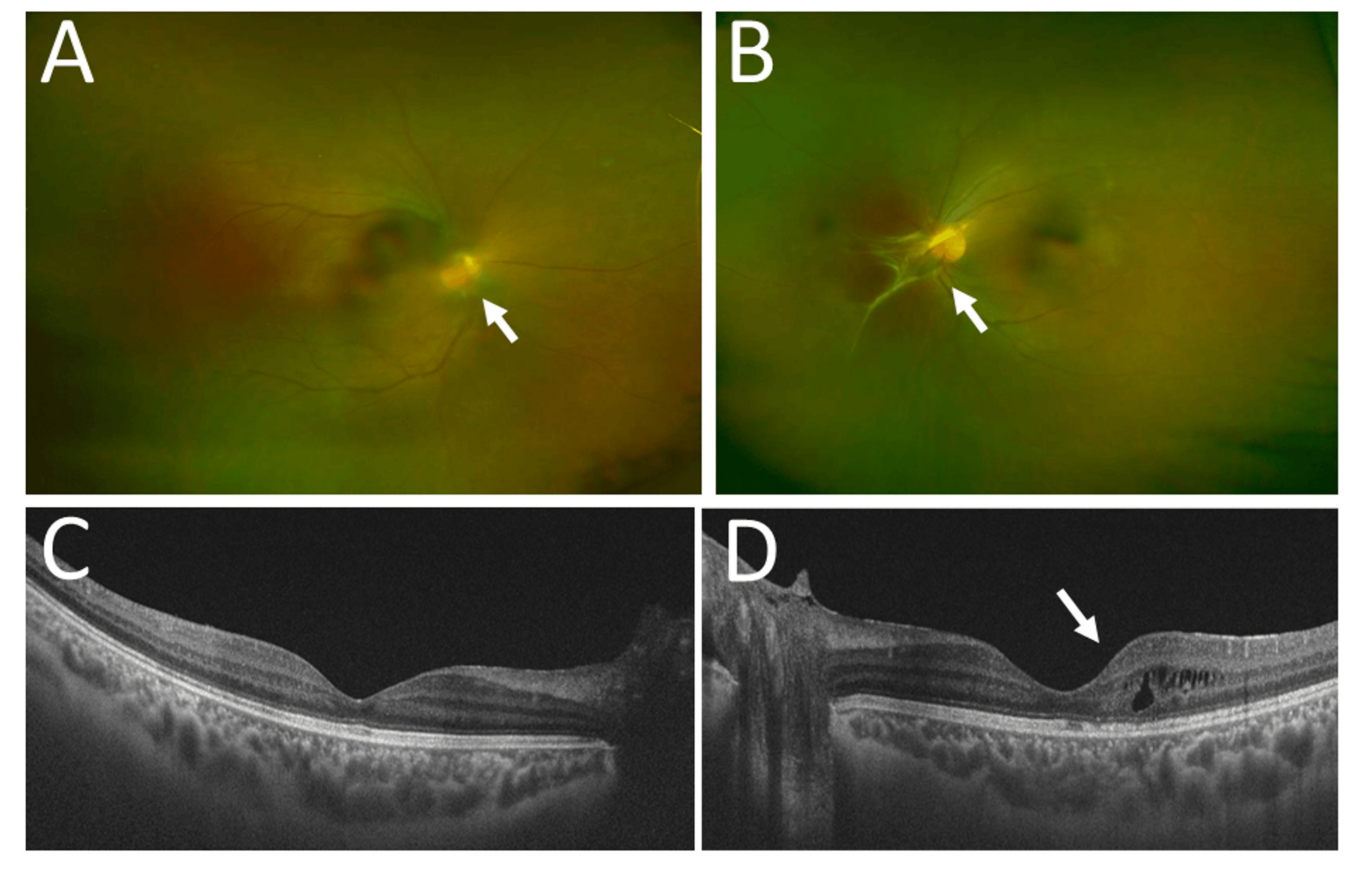
Clinical images in Case 16 (female) at 21 years of age Wide-field fundus photographs (A: right eye; B: left eye), showing residual white tissue on optic discs (arrows). Horizontal section images of optical coherence tomography (C: right eye; D: left eye), showing mildly disrupted photoreceptor ellipsoid zones with residual intraretinal fluids only in the left eye (arrow).

Oral prednisolone was discontinued after tapering to 5 mg every other day at the age of 16 years, one and a half years after the initial visit. She continued topical betamethasone twice daily, and half a year later, she began using topical 0.1% bromfenac twice daily in combination with topical betamethasone once daily. One year later, she discontinued topical betamethasone and used topical bromfenac twice daily alone. She remained stable in these processes with no inflammation until the age of 17 years, three years after the initial visit, when she experienced a second relapse of cystoid macular edema in the left eye. At that time, she began oral colchicine 1 mg daily instead of oral prednisolone, together with topical bromfenac twice daily, resulting in the resolution of macular edema in the left eye within one month. The dose of oral colchicine was reduced to 0.5 mg daily and discontinued after one and a half years at the age of 19 years, while she continued to use topical bromfenac twice daily. One year later, at the age of 20 years, she delivered her first baby without complications during pregnancy and delivery. She had no major relapse while using topical betamethasone twice daily and topical bromfenac twice daily until the age of 23 years (Figure 6).

Case 17

**Figure 7:**
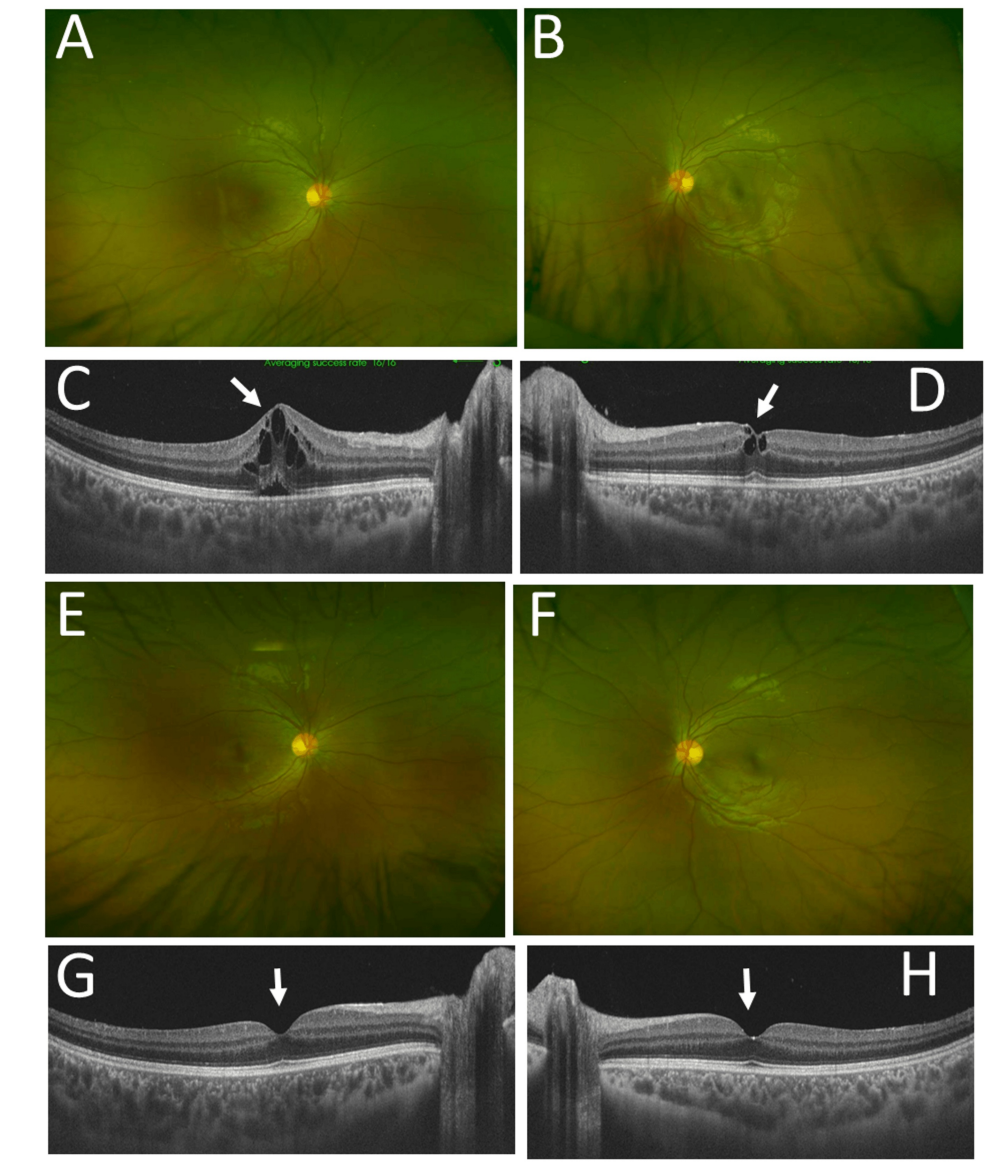
Clinical images in Case 17 A 15-year-old female. Wide-field fundus photographs (A: right eye; B: left eye) and horizontal section images of optical coherence tomography (C: right eye; D: left eye), showing macular edema (arrows). Macular edema in both eyes (E, G: right eye; F, H: left eye) has subsided (arrows) six weeks later with oral prednisolone tapering from 30 mg daily.

A 15-year-old female noticed blurred vision in both eyes at school after the summer vacation. She was diagnosed with bilateral iridocyclitis, prescribed topical 0.1% betamethasone eye drops four times daily, and referred to a university hospital. At referral, the best-corrected visual acuity was 1.0 in both eyes, and the intraocular pressure was 16 mmHg in both eyes. She showed 1+ mutton-fat keratic precipitates with no aqueous cells and 1+ vitreous cells in both eyes. Iris nodules as well as anterior and posterior iris synechiae were absent. The optic discs in both eyes were mildly hyperemic, and optical coherence tomography showed macular edema in the right eye and macular cysts in the left eye (Figure 7, Panels A-D). She had no past medical history and no systemic symptoms or signs, including skin rashes and fever. Blood examinations, including complete blood cell counts, blood chemistry, and glucose, as well as a plain chest X-ray film, were all normal. Serological tests for syphilis, hepatitis B virus surface antigen, and hepatitis C virus antibody were all negative. Urinalysis, including measurement of NAG, was also normal. She began oral prednisolone 30 mg daily, which was tapered to 20 mg daily. After one month, she had no macular edema in both eyes (Figure 7, Panels E-H) but showed elevated intraocular pressure to 25 mmHg in both eyes because she was a steroid responder. The dose of oral prednisolone was therefore reduced to 5 mg daily, and topical 0.1% betamethasone four times daily was replaced with topical 0.1% bromfenac four times daily.

Within two weeks, the intraocular pressure in both eyes returned to normal. Two months after the initial visit, she experienced a relapse of macular edema in the right eye while receiving oral prednisolone 5 mg every other day. The dose of oral prednisolone was increased to 20 mg every other day. After one month, residual macular edema persisted in the right eye, and she complained of a moon face. At that time, oral colchicine 1 mg daily was introduced, and oral prednisolone was reduced to 10 mg every other day, resulting in the resolution of macular edema in both eyes within one month. Half a year later, 11 months after the initial visit, oral prednisolone 5 mg every other day was discontinued, and oral colchicine 1 mg daily was continued together with topical 0.1% bromfenac four times daily. Oral colchicine was reduced to 0.5 mg daily one year later, two years after the initial visit, and was discontinued another year later, three years after the initial visit. She showed no relapse until the last visit at the age of 18 years.

Case 18

**Figure 8:**
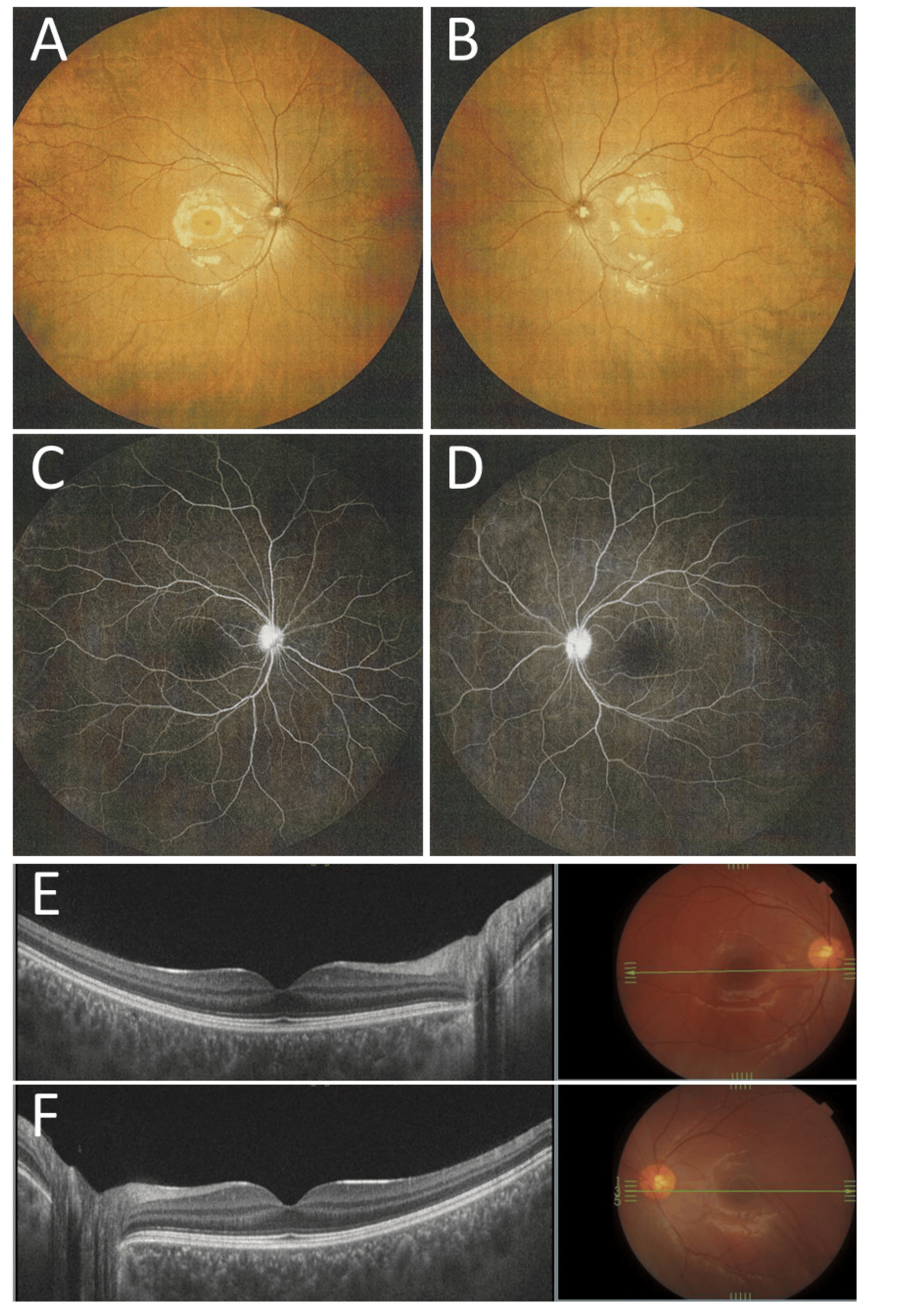
Clinical images in Case 18 A 12-year-old female. Wide-field fundus photographs (right eye: A; left eye: B) and fluorescein angiograms (right eye: C; left eye: D), showing hyperemic optic discs and dye leakage from the optic discs in both eyes. Horizontal section images of optical coherence tomography on the left panels and fundus photographs on the right panels (right eye: E; left eye: F), with no oral medication at 13 years of age, 7 months from the disease onset.

A 12-year-old female noticed photophobia and redness in the left eye and visited a hospital. The best-corrected visual acuity was 1.2 in both eyes, and the intraocular pressure was 15 mmHg in the right eye and 18 mmHg in the left eye. Slit-lamp examination showed 1+ mutton-fat keratic precipitates, 2+ aqueous cells, and 2+ anterior vitreous cells in both eyes. Fundus examination showed hyperemic optic discs in both eyes with cloudy peripheral retina (Figure 8, Panels A and B), and fluorescein angiograms revealed retinal capillary leakage in the peripheral fundus of both eyes with optic disc leakage (Figure 8, Panels C and D). Systemic evaluation by a pediatrician revealed no abnormalities. Because of hyperemic optic discs and severe photophobia in both eyes, she began oral prednisolone tapered from 30 mg daily, together with topical 0.1% betamethasone four times daily. In two weeks, the intraocular pressure increased to 29 mmHg in both eyes. As she was diagnosed as a steroid responder, topical betamethasone was replaced with topical 0.1% fluorometholone four times daily in both eyes, and oral prednisolone was tapered at a fast pace. Three months after the onset, she experienced a relapse of iridocyclitis in both eyes while receiving oral prednisolone 12.5 mg daily and began oral methotrexate 6 mg weekly in addition to oral prednisolone 17.5 mg daily. Six months after onset, she was receiving oral prednisolone 5 mg daily and methotrexate at a maximum dose of 16 mg weekly, together with a topical combination of 1% dorzolamide and 0.5% timolol. She had persistent general fatigue, and her family wished to be consulted at a university hospital.

At referral, seven months after the onset, the best-corrected visual acuity was 1.5 in both eyes, and the intraocular pressure was 15 mmHg in both eyes. She showed a small number of mutton-fat keratic precipitates and aqueous cells, together with normal maculae and normally colored optic discs in both eyes (Figure 8, Panels E and F). In consultation with a pediatrician, oral methotrexate was discontinued because her general fatigue was considered to be related to methotrexate overdose. Oral prednisolone 5 mg daily was changed to 5 mg every other day. She began topical 0.1% bromfenac four times daily in addition to a topical combination of 1% dorzolamide and 0.5% timolol. In one month, she felt healthy without oral medication and showed 1+ mutton-fat keratic precipitates in both eyes. She began using topical 0.1% betamethasone once daily and did not develop elevated intraocular pressure in both eyes. During 2.5 years of follow-up until the latest visit at the age of 15 years, she used topical 0.1% betamethasone once or twice daily according to her symptoms. The intraocular pressure remained within the normal range in both eyes, with minimal keratic precipitates and no aqueous cells.

Summary of 18 cases

The 18 patients comprised 6 males and 12 females, with the age at onset ranging from 9 to 16 years (median, 13 years) (Table 1). All patients presented with aqueous cells and mutton-fat keratic precipitates in both eyes, and fluorescein angiography revealed varying degrees of optic disc leakage and retinal capillary leakage in the midperipheral to peripheral fundus of both eyes. They had no systemic symptoms, including skin rashes, joint pain, or fever, and showed no abnormalities on blood examinations, urinalysis, or plain chest X-ray films. Oral prednisolone tapered from 30 mg daily, together with topical 0.1% betamethasone four times daily, was administered to five patients: two with optic disc edema (Cases 11 and 18) and three with macular edema in both eyes (Cases 16 and 17) or in the unilateral eye (Case 7). One of these five patients (Case 16) also showed notable optic disc edema in combination with marked macular edema in both eyes. The remaining 13 patients were treated with topical 0.1% betamethasone eye drops alone. In patients with elevated intraocular pressure, often because they were steroid responders, the instillation of topical 0.1% betamethasone was reduced to twice daily, or intraocular pressure-lowering eye drops were used in combination. In two patients (Cases 16 and 17) who experienced a relapse of unilateral macular edema after oral prednisolone was tapered or discontinued, oral colchicine 1 mg daily was introduced, resulting in the subsidence of macular edema. Bilateral uveitis in all 18 patients subsided within half a year to a few years until 19 years of age, and topical medications were discontinued without relapse except for one patient (Case 16).

**Table 1:** Clinical features in 18 patients with BIRC in juveniles “One visit” in follow-up indicates only one referral visit for consultation to confirm the diagnosis and treatment. Visual acuity was measured in decimals. Aqueous cells and KP were present only in the left eye at the initial visit and in the right eye at relapse 4 years later in Case 10. BIRC: Bilateral iridocyclitis with retinal capillaritis; KP: Mutton-fat keratic precipitates.

Case no./Gender	Age at onset (years)	Follow-up periods (years)	Best-corrected visual acuity at initial visit, right eye/left eye	Best-corrected visual acuity at final visit, right eye/left eye	Anterior segment manifestations in both eyes at the initial visit	Posterior segment manifestations in both eyes at the initial visit	Topical corticosteroids	Oral corticosteroids and other medications	No. of relapse, age at remission and no treatment
1/Female	9 years	One visit	1.0/1.0	Not applicable	Aqueous cells, KP	Midperipheral edema	Betamethasone 0.1%	No	Not known
2/Female	12 years	12 years	1.5/1.5	1.5/1.5	Aqueous cells, KP	Midperipheral edema	Betamethasone 0.1%	No	Once, 18 years
3/Male	13 years	One visit	1.0/1.0	Not applicable	Aqueous cells, KP	Midperipheral edema	Betamethasone 0.1%	No	Not known
4/Female	13 years	One visit	1.0/1.0	Not applicable	Aqueous cells, KP	Midperipheral edema	Betamethasone 0.1%	No	Not known
5/Female	14 years	One visit	1.0/1.0	Not applicable	Aqueous cells, KP	Midperipheral edema	Betamethasone 0.1%	No	Not known
6/Female	10 years	One visit	1.2/1.2	Not applicable	Aqueous cells, KP	Midperipheral edema	Betamethasone 0.1%	No	Once, no data on age
7/Female	15 years	One visit	1.0/1.0	Not applicable	Aqueous cells, KP	Midperipheral edema, right macular edema	Betamethasone 0.1%	Prednisolone 30 mg daily and tapering	Not known
8/Male	13 years	One visit	1.0/1.0	Not applicable	Aqueous cells, KP	Midperipheral edema	Betamethasone 0.1%	No	Not known
9/Male	16 years	One visit	1.0/1.0	Not applicable	Aqueous cells, KP	Midperipheral edema	Betamethasone 0.1%	No	Not known
10/Female	13 years	29 years	1.5/1.5	1.5/1.5	Aqueous cells, KP	Midperipheral edema	Betamethasone 0.1%	No	Once, 19 years
11/Male	16 years	2 years	1.5/1.5	1.5/1.5	Aqueous cells, KP	Midperipheral edema, bilateral optic disc edema	Betamethasone 0.1%	Prednisolone 30 mg daily and tapering	Twice, 18 years
12/Male	13 years	6 years	1.5/1.2	1.2/1/2	Aqueous cells, KP	Midperipheral edema	Betamethasone 0.1%	No	Once, 18 years
13/Female	13 years	5 years	1.0/1.0	1.5/1.5	Aqueous cells, KP	Midperipheral edema	Betamethasone 0.1%	No	Once, 18 years
14/Female	14 years	4 years	1.0/1.0	1.5/1.5	Aqueous cells, KP	Midperipheral edema	Betamethasone 0.1%	No	Once, 18 years
15/Male	14 years	3 years	1.5/1.5	1.5/1.5	Aqueous cells, KP	Midperipheral edema	Betamethasone 0.1%	No	Once, 17 years
16/Female	14 years	10 years	0.8/0.8	0.5/0.5	Aqueous cells, KP	Midperipheral edema, bilateral macular edema, bilateral optic disc edema	Betamethasone 0.1%	Prednisolone 30 mg daily and tapering, Colchicine 1 mg daily	Twice, ongoing treatment
17/Female	15 years	3.5 years	0.9/1.0	1.2/1.2	Aqueous cells, KP	Midperipheral edema, bilateral macular edema	Betamethasone 0.1%	Prednisolone 30 mg daily and tapering, Colchicine 1 mg daily	Once, 18 years
18/Female	12 years	2.5 years	1.2/1.5	1.5/1.5	Aqueous cells, KP	Midperipheral edema, bilateral optic disc edema	Betamethasone 0.1%	Prednisolone 30 mg daily and tapering, Temporary weekly methotrexate	Once, ongoing treatment

## Discussion

In a previous study [4], the clinical features of 18 consecutive patients diagnosed with BIRC in juveniles over 11 years (1985-1995) were described. The 18 patients included 6 males and 12 females, with an age at onset ranging from 9 to 17 years (median, 13 years). They presented with blurred vision, photophobia, and redness, while all had a best-corrected visual acuity of 20/20 (1.0 in decimals) or better. Slit-lamp examination showed mutton-fat keratic precipitates, aqueous cells, and anterior vitreous cells in both eyes as the manifestations of iridocyclitis. At the initial visit, a few patients showed anterior or posterior iris synechiae as sequelae of chronic iridocyclitis. Fundus examination revealed hyperemic or normal optic discs in both eyes with cloudy midperipheral to peripheral retina with edema to varying extents, sometimes associated with low-grade macular edema. Fluorescein angiography revealed dye leakage from the optic discs together with leakage from retinal capillaries in the peripheral-to-midperipheral fundus of both eyes, corresponding to the cloudy retina. Other retinal manifestations included spotty retinal exudates, segmental retinal periphlebitis on rare occasions, and snowball-like vitreous opacities after relapse. No systemic abnormalities were detected by plain chest X-ray films, blood examinations, or urinalysis in consultation with a rheumatologist or pediatrician.

The patients in the previous study [4] were treated with topical corticosteroid eye drops alone or in combination with oral or intravenous prednisolone based on the extent of iridocyclitis and retinal inflammation. All patients responded well to topical and systemic corticosteroids and experienced one or two relapses of iridocyclitis over several years. Subsequently, they remained relapse-free and discontinued corticosteroid eye drops. No systemic manifestations developed during follow-up.

In the present study, 18 additional consecutive patients diagnosed over the subsequent 29-year period (1996-2024) included 6 males and 12 females, with an age at onset ranging from 9 to 16 years (median, 13 years). Comparison of the previous [4] and present studies showed the same male-to-female ratio (1:2), indicating female predominance, and the same median age at onset of 13 years within the school-age to early adolescent period.

Regarding the treatment, 13 of the 18 patients in the present study were managed with topical corticosteroid eye drops alone, primarily 0.1% betamethasone, compared with 5 of the 18 patients in the previous study [4]. Following publication of the previous study [4], the newly recognized entity of juvenile uveitis, BIRC, became well-known in the community of ophthalmologists in this area, and the number of consultations increased in regard to the diagnosis of BIRC and the treatment strategies. Consequently, 8 of the 18 patients in the present study visited only once for the consultations. These patients showed mild iridocyclitis and mild peripheral-to-midperipheral retinal capillaritis at referral, which responded to topical 0.1% betamethasone eye drops alone. During consultation, the disease course, including the possibility of one or two relapses and the overall good prognosis with remission by approximately 18 years of age, was explained to the patients and their families. Treatment strategies, including adjustment of the daily frequency of topical betamethasone based on the extent of iridocyclitis and steroid responsiveness, were also discussed.

Consequently, only a limited number of patients continued follow-up at the university hospital. In addition, most patients were lost to follow-up after 18 years of age because they moved to other areas for higher education, employment, or marriage. Therefore, the long-term outcome after disease subsidence remains to be determined. As an exception, one patient (Case 10) continued follow-up because she remained in the local area and wished to be monitored after the subsidence of iridocyclitis without treatment. She showed no relapse for up to 20 years after the subsidence. Although this observation is limited to a single patient, it suggests that BIRC in juveniles does not relapse in adulthood.

Regarding visual prognosis, only one patient (Case 16) showed not-the-best optimal visual acuity after the subsidence of iridocyclitis with macular edema in both eyes. This patient showed marked leakage from the optic discs and retinal capillaries extending widely into the posterior pole (Figure 5) and was treated with oral prednisolone 30 mg daily followed by tapering. In retrospect, high-dose intravenous prednisolone might have been a better treatment option for preserving vision. Her adherence to topical betamethasone was poor and might result in residual inflammation, leading to mildly interrupted photoreceptor ellipsoid zones in macular areas of both eyes.

As a treatment option, low-dose oral colchicine (1 mg or 0.5 mg daily) was used alone or in combination with oral prednisolone in two patients (Cases 16 and 17) who developed recurrent macular edema during tapering or after discontinuation of oral prednisolone. Because oral prednisolone administered daily for one month or longer commonly causes moon-face appearance, young adolescents are often reluctant to continue long-term treatment. In addition, oral prednisolone for a long term also influences growth in young adolescents. In this context, cyclosporine is an alternative immunosuppressive agent approved for the treatment of uveitis, but its use is limited by its slower onset of action and more frequent adverse effects compared with short-term corticosteroids. Colchicine has been used for more than half a century and has been shown to be safe and effective for the treatment of uveitis associated with Behçet disease [13]. In the present study, recurrent macular edema was successfully controlled in two recent patients (Cases 16 and 17) who received colchicine at 1 mg daily, tapered to 0.5 mg daily, and eventually discontinued. In other fields of medicine, low-dose colchicine has also been used as an anti-inflammatory adjunct in the treatment of myocarditis and myopericarditis [15-17].

Case 18 illustrates the importance of adjusting the frequency of topical 0.1% betamethasone instillation in patients who develop elevated intraocular pressure in response to topical and oral corticosteroids (steroid responders) [18]. The previous ophthalmologist discontinued topical 0.1% betamethasone and instead prescribed weekly oral methotrexate together with low-dose oral prednisolone. The diagnosis may have been juvenile chronic iridocyclitis in young girls [14], and methotrexate was prescribed weekly at a high dose according to the standard treatment for juvenile idiopathic arthritis. However, the patient did not tolerate the high weekly dose of methotrexate, and she and her family sought consultation with another ophthalmologist specializing in uveitis. Topical 0.1% betamethasone was resumed twice daily, and intraocular pressure was monitored to determine the maximum tolerable duration and frequency of instillation. It should be emphasized that topical 0.1% betamethasone, unlike 0.1% fluorometholone, reaches the retina and is therefore effective in reducing macular edema and optic disc edema [19]. Topical 0.1% bromfenac, a nonsteroidal anti-inflammatory drug, also reaches the retina [19] and may be used in combination with topical betamethasone. Long-term topical betamethasone therapy in young patients would not induce steroid cataract, whereas long-term lingering iridocyclitis which is not controlled well with topical corticosteroids is more likely to result in the development of complicated cataract.

It should be noted that, in both the present study and the previous study [4], no patient developed systemic symptoms or signs during follow-up that suggested systemic diseases such as juvenile idiopathic arthritis or tubulointerstitial nephritis. In most patients in the present series, especially in the early half of the period, 24-hour urine collections were used to measure total protein, NAG, and β2-microglobulin. In several recent patients, these markers were measured in spot urine because their assessment has become more reliable in recent years. Tubulointerstitial nephritis [8] and inflammatory bowel disease [9] may show similar manifestations of retinal capillary leakage on fluorescein angiography. Ulcerative colitis and Crohn's disease typically present with symptoms such as bloody or mucoid stools, abdominal pain, and diarrhea, whereas tubulointerstitial nephritis may be asymptomatic. Therefore, urinalysis to exclude tubulointerstitial nephritis at the initial presentation of juvenile uveitis is essential in the differential diagnosis. Juvenile chronic iridocyclitis in young girls, which shares similar eye manifestations with juvenile idiopathic arthritis-associated uveitis, appears to represent a distinct entity because its onset typically occurs in preschool-aged children [14], whereas BIRC occurs in school-aged children and young adolescents.

In the current ophthalmic practice, optical coherence tomography plays a crucial role in detecting macular changes such as macular edema and has largely replaced fluorescein angiography. Fluorescein angiography carries a risk of allergic reactions and is relatively difficult to perform in children, whereas optical coherence tomography is not time-consuming and does not put a load on examinees. In the diagnosis and follow-up of BIRC, detection of macular edema is essential for preserving vision and guiding treatment decisions, including adjustment of topical betamethasone frequency and initiation or resumption of oral prednisolone or colchicine. Therefore, from a patient-centered perspective, fluorescein angiography may be omitted in the diagnosis of BIRC in current clinical practice.

## Conclusions

This study reports the clinical features of 18 additional patients with BIRC in juveniles following its initial description in 18 patients in the author's previous report. Comparison of the previous and present BIRC cohorts showed the same male-to-female ratio of 1:2 and the same median age at onset of 13 years within the school-age and early adolescent age groups. The patients presented with bilateral iridocyclitis characterized by mutton-fat keratic precipitates, aqueous cells, and anterior vitreous cells, together with hyperemic optic discs and retinal cloudiness of varying severity. They commonly presented with blurred vision and photophobia, although visual acuity remained within the normal range. All 18 patients in the present study, as well as the 18 patients in the previous study, showed a good response to topical or systemic corticosteroids, had a favorable outcome with one or two relapses over a few years, and resolved by late adolescence without progression to systemic autoimmune disease. Follow-up with optical coherence tomography is the best way to detect macular edema over the course of the disease and determine whether oral prednisolone or colchicine may be required.

Urinalysis at the initial presentation is crucial to exclude tubulointerstitial nephritis because this condition may not present with systemic symptoms. Differential diagnoses include infectious uveitis caused by syphilis and tuberculosis and non-infectious uveitis associated with juvenile idiopathic arthritis (JIA), juvenile chronic iridocyclitis (JCI) in young girls, which is associated with JIA, tubulointerstitial nephritis, inflammatory bowel disease, and Behçet disease. The entity of BIRC in juveniles would help ophthalmologists, patients, and their families expect a better outcome and understand how to cope with uveitis in a few years. Appropriate adjustment of the daily frequency of topical betamethasone instillation is key to managing iridocyclitis, particularly in patients with associated macular edema or, less commonly, optic disc edema. Optical coherence tomography is useful for detecting macular edema during the diagnosis and follow-up of BIRC and for guiding decisions regarding the initiation of oral prednisolone or colchicine.
